# Risk of root damage after using lateral cephalogram and intraoral scan for guided insertion of palatal miniscrews

**DOI:** 10.1186/s13005-022-00335-0

**Published:** 2022-09-03

**Authors:** Manuel Nienkemper, Björn Ludwig

**Affiliations:** 1 University of Düsseldorf, Düsseldorf, Germany; 2 University of Homburg, Traben-Trarbach, Germany

**Keywords:** Skeletal anchorage, Root damage, Guided insertion, Imaging

## Abstract

**Background:**

Guided insertion of palatal miniscrews using a lateral cephalogram instead of cone beam computed tomography (CBCT) significantly reduces the radiation level for the patient. Till now no data are available on the risk of hitting the incisors in this regard, which is one of the worst clinical complications when inserting a paramedian miniscrew. Hence, this study aims to investigate the distance between the mini-implant and the roots of the central and lateral incisors.

**Methods:**

Lateral cephalogram, an intraoral scan, and CBCT of 20 patients were superimposed. After a miniscrew (1.7 × 8 mm) placement based on intraoral scan and lateral cephalogram, the CBCT was used as control for the distance between the miniscrews and the roots of the incisors.

**Results:**

The mean value of the shortest distance between the miniscrew and roots of the incisors in the lateral cephalogram was 4.74 ± 1.67 mm. The distance between both miniscrews and the central incisors measured in the CBCT was 5.03 ± 2.22 mm and 5.26 ± 2.21 mm and between the two miniscrews and the lateral incisors was 4.93 ± 1.91 mm and 5.21 ± 2.64 mm. No significant differences between the distances in the CBCT and the lateral cephalogram could be observed. In one case, the CBCT control revealed the penetration of two palatally displaced canines after insertion based on intraoral scan and lateral cephalogram.

**Conclusions:**

The use of an intraoral scan and a lateral cephalogram for guided paramedian insertion of palatal miniscrews can prevent incisor root damage. This may reduce the radiation since no CBCT seems necessary. The current investigation focuses on the anterior paramedian area of the palate. Outside that region and in complex cases with displaced teeth in the palatal area, a CBCT might be indicated.

## Background

Over the past two decades, various approaches for skeletal anchorage have been developed [1, 2, 3]. Within this group, orthodontic mini-implants have become the most common variant [1, 4, 5, 6]. They have proven to reinforce orthodontic anchorage in clinical practice [7, 8] and have opened up new treatment options [9, 10]. The literature shows that the success rates are encouraging [11, 12, 13]. In particular, the anterior palate has been established as the insertion region because of its good bone supply [14, 15]. Taking a closer look, the ideal spot can be found within a T-shaped area, anterior paramedian, and median along the suture [16]. Studies have shown high stability after insertion in this area, with no statistical differences for median and paramedian implants [17, 18]. The paramedian area can be used for nearly all indications, including skeletal borne rapid palatal expansion (RPE), hence it is preferred by most clinicians [10, 19]. The best clinical guideline to identify the paramedian area of the T-Zone appears to be the third palatal ruga, whereas dental landmarks are not reliable due to possible tooth migration [20]. One drawback of paramedian insertion compared to median placement is its closer proximity to the incisor roots. On one hand, the distance to the dental roots is correlated with the success rate of mini-implants [21, 22] and on the other hand, there is a risk of root damage. The superficial lesion seemed to heal with the cementum [23]. When the mini-implant hits the apex of the tooth, the bundle of nerves and blood vessels can be cut, and root canal treatment must be performed [24]. For precise and safe insertion of dental implants, the use of a surgical guide was introduced a long time ago [25]. Currently, it is well established, mostly based on CBCT data, and its accuracy has been underlined in current investigations [26]. Orthodontic mini-implants are mostly inserted without guidance. However, a paper published in 2009 by Cousley introduced a surgical guide for mini-implant insertions [27]. Maino et al. were the first to superimpose a lateral cephalogram with an intraoral scan of the maxilla for mini-implant planning [28]. Kim et al. measured the bone height at the anterior palate on the CBCT median and stepwise to the sides, and compared it with the bone height measured on the lateral cephalogram [29]. They found that the bone height seen on the lateral cephalogram matched the bone height 5 mm paramedian on CBCT. Between the 5 mm paramedian and suture, the bone height was even higher. Since the lateral cephalogram underestimates the paramedian bone supply close to the midline, it seems suitable to use the superimposition of intraoral scan and lateral cephalogram for paramedian-guided insertion within 5 mm from the midline. This would make CBCT superfluous in these cases, leading to a significant reduction in the radiation dose for patients. The results of the first study support the applicability of this protocol with regard to bone support [30]. There are no data available on the risk of hitting the incisors in this regard, which is one of the worst clinical complications when inserting a paramedian miniscrew. Hence, this study aims to investigate the distance between the mini-implant and the roots of the central and lateral incisors. The risk of root damage after using lateral cephalograms and intraoral scans for the guided insertion of palatal miniscrews should be evaluated.

## Methods

The patient database of a private practice in Traben-Trarbach, Germany, was screened. The inclusion criteria were initial diagnostics containing a lateral cephalogram, an intraoral scan, and CBCT. Since, CBCT is not a standard diagnostic tool in orthodontics, in most cases, it was performed with a certain time lag to elucidate special findings. For inclusion in this study, the maximum time period between the scan, lateral cephalogram, and CBCT was set to 2 weeks. The exclusion criteria were cleft and impacted upper incisors.

**Fig. 1:**
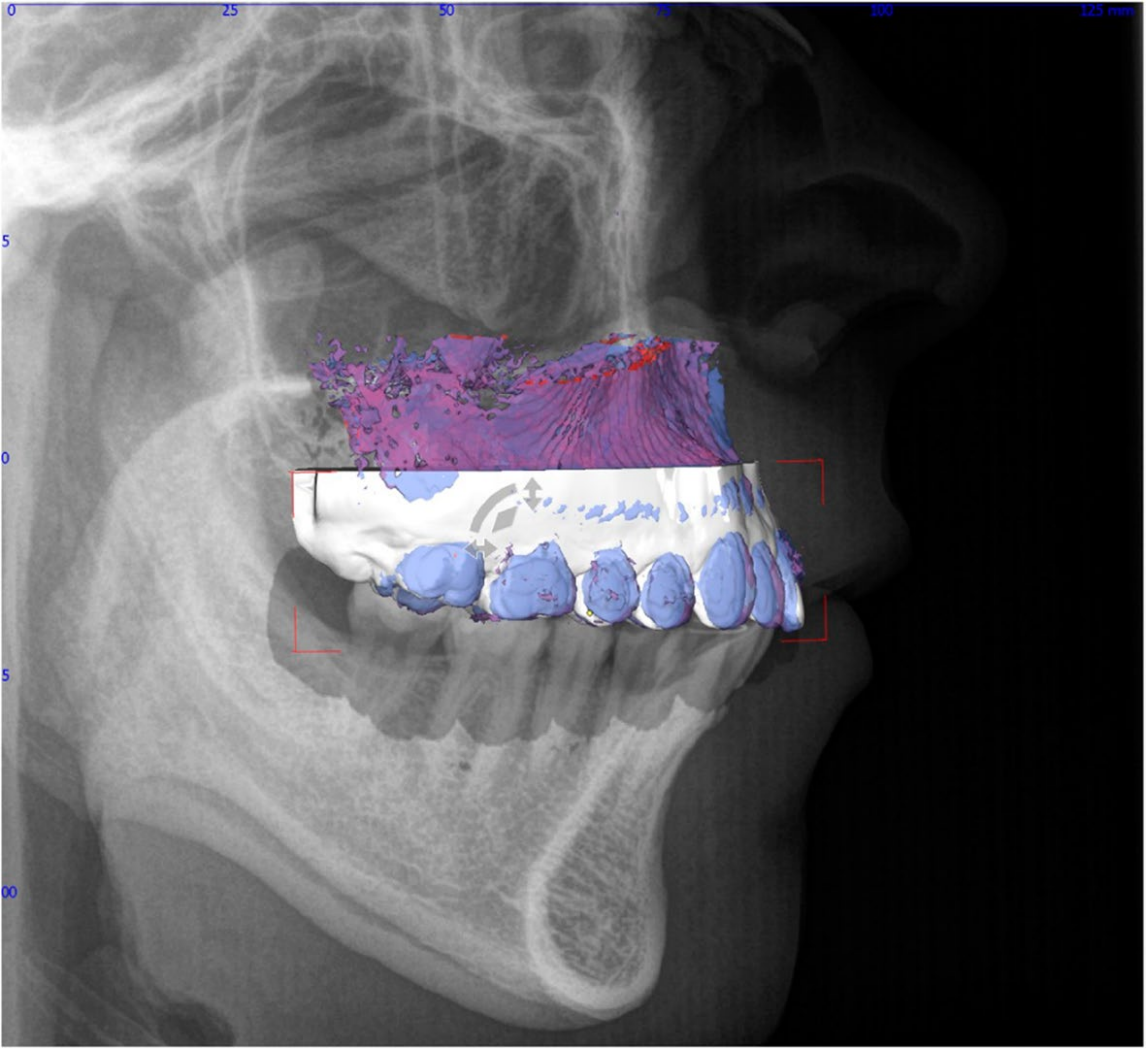
Data set after three-dimensional superimposition of lateral cephalogram, intraoral scan of the maxilla, and CBCT

For each patient, the lateral cephalogram, intraoral scan of the maxilla, and CBCT were superimposed using OnyxCeph^3^™® software (image instruments, Chemnitz, Germany) (Fig. 1). Incisal edges of the central incisors and the buccal cusp tips of the molars were used for matching in the YZ-plane. To superimpose 2D and 3D data according to the XY-plane, a specially developed algorithm was used [31].

**Fig. 2:**
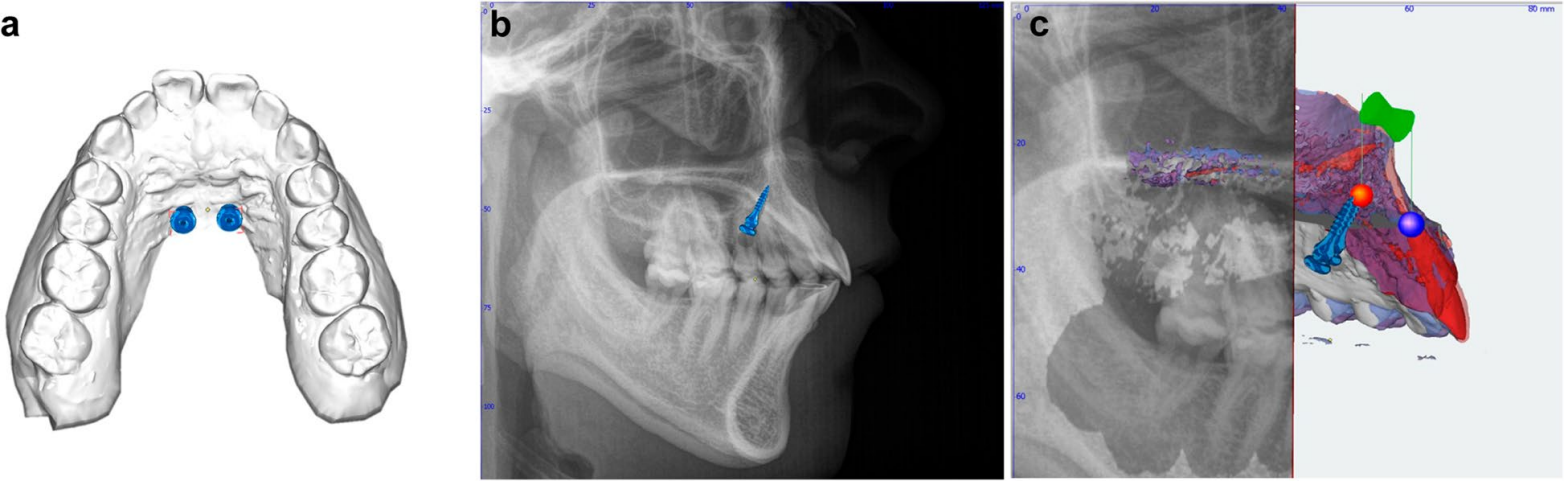
Sequence of insertion and measurements. Placement according to clinical guidelines (**a**) correction of position and angle using the lateral cephalogram, followed by measurement of the distance between miniscrews and incisors (**b**) application of the CBCT for measurement of the distances between both implants and central and lateral incisors

First, the intraoral scan of the maxilla was selected and two mini-implants (1.7 × 8 mm, Orthoeasy, Forestadent, Germany) were inserted at the anterior palate, according to clinical guidelines, directly behind the third palatal rugae and 3 mm paramedian (Fig. 2a). Subsequently, the matched lateral cephalogram was displayed, and the implant position was optimised according to the bone supply and distance to the incisor roots (Fig. 2b). After that, the CBCT was displayed as reference, and the shortest distance between each mini-implant and the respective lateral and central incisors was measured using OnyxCeph^3^™® software (Fig. 2c). These values were compared to each other and to the shortest distance between the mini-implant and the incisors on the lateral cephalogram.

All data were tested for normal distribution using the Shapiro-Wilk test. All measurements were taken twice by a second investigator to evaluate the reproducibility of distance measurements. Paired t-tests (*p* < 0.05) were used to compare measurements. All distances were tested for statistical differences using ANOVA. For an additional assessment of the setting value, 95% confidence intervals were calculated. Descriptive statistics were used to examine single values of special interest.

## Results

Twenty patients were included in the study after applying inclusion and exclusion criteria. The group consisted of nine female and 11 male patients. The mean age was 13.95 ± 3.40 years. Since the Shapiro-Wilk test revealed a normal distribution of the data, parametric tests were applied.

The paired t-tests showed no statistical significance between the first and repeated measurements; *p*-values ranged from 0.193 to 0.821.

ANOVA showed no differences (*p* < 0.05) in the distances between the incisors and implants in the lateral cephalograms and CBCT images (Table 1). The mean values even showed a shorter distance measured in the lateral cephalogram than that measured for each incisor using CBCT. However, these differences were not statistically significant. The extensive overlap of all confidence intervals underlines the value of the chosen settings of the study design.

**Table 1:** Distances between miniscrews and roots measured in the lateral cephalogram and in the CBCT

		Distance	SD	95% confidence interval		minimum
TAD	lateral ceph	4.74	1.67	3,96 -5,61		2.1
TAD right	CBCT 12	4.93	1.91	4,03 - 5,82		1
TAD right	CBCT 11	5.03	2.22	3,99 - 6,07		0.2
TAD left	CBCT 21	5.26	2.21	4,22 - 6,29		1.5
TAD left	CBCT 22	5.22	2.64	3,98 - 6,45		1.3
	ANOVA	n.s.				

Looking at the descriptive data, the minimum distance measured in each group was slightly smaller than the distances measured using CBCT. The shortest distance was found between the right temporary anchorage device (TAD) and right central incisor (0.2 mm).

In one case, the evaluation of the implant position using CBCT revealed the penetration of two palatally impacted canines.

## Discussion

The anterior palate, especially the paramedian area, has become one of the most favoured sites for mini-implant insertion [18, 32]. To make insertions more secure and to enable insertion of implants and appliances in one visit, guided mini-implant insertion is becoming increasingly popular [33]. The replacement of CBCT with a lateral cephalogram for 3D planning of paramedian mini-implants would lead to a significant reduction in the radiation dose for the patient. A lateral cephalogram is normally taken when planning a case; therefore, no extra X-ray would be needed for implant planning. This seems to be sufficient for the evaluation of bone support [29, 30]. The distance to the roots correlates with the success rate of the mini-implants [21]. Moreover, since severe damage to an incisor root is a realistic scenario [24] this method should be investigated in this regard. This was the aim of this study.

On the lateral cephalogram, there was a superimposition of all four incisors in one layer. Owing to the 2-dimesional recording technique, not all roots can be seen clearly. After using the combination of intraoral scan and lateral cephalogram for implant insertion, the real distances of each implant to the respective central and lateral incisors in CBCT were measured three-dimensionally as a control.

For this purpose, a group of patients was collected for whom an intraoral scan, lateral cephalogram, and CBCT were available. According to the inclusion criteria, CBCT was performed not later than 2 weeks after the basic diagnosis to ensure that no changes occurred. The software used for superimposition and measurements has already been proven to be applicable to other investigations [34]. The method for superimposing the two-dimensional lateral cephalogram and the three-dimensional data, according to the XY-plane was subject to a critical investigation [31]. The reproducibility of the measurements was proven by the repeated performance, which showed no significant differences for all measurements. For the clinical relevance of this study it is crutial, that the virtually planned position of the TAD is achievable with clinical precision. This was affirmed by an investigation of Iodice et al. [35].

Considering the mean values and standard deviations, the results showed safe distances to the roots in all groups. There were no statistically significant differences between the distances measured in the lateral cephalogram and those measured using CBCT in all groups. The extensive overlap of all 95%confidence intervals underlines the value of the chosen settings of the study, indicating a sufficient number of cases. The distances seen in the lateral cephalogram even underestimated the “real” distances in the CBCT. These findings underline the applicability of a combination of intraoral scans and lateral cephalograms. Using CBCT as a control, no incisor root was hit by the implant. Taking a closer look at the minimum distances found in each group, the values were slightly smaller in the CBCT groups, but did not go below 1 mm, with one exception. A single root showed a distance of 0.2 mm. In addition to the fact that this might affect the survival of mini-implants [21] it might also lead to minor damage to the root. Maino et al. intentionally moved bicuspids towards mini-implants and reported that minor resorptions in some cases already occurred at a distance of 0.6 mm [36]. However, these small lesions healed with cementum. Moreover, attention should be paid to the fact that in one case, the palatally impacted canines were hit by mini-implants when evaluating their position on CBCT.

Overall, the results of this study indicate that the use of an intraoral scan and a lateral cephalogram for guided paramedian insertion of mini-implants can prevent incisor root damage. No potential damages of the incisal roots were observed. The distance in the lateral cephalogram underestimated the real distances on average. However, a safe distance should be maintained because avoidance of very close proximity is not ensured in every single case. It should also be mentioned, that this protocol is strictly limited to the anterior palate within the T-Zone. CBCT should be performed outside this area and in complex cases of displaced canines.

## Conclusions

Based on the results of this investigation it can be concluded that the use of an intraoral scan and a lateral cephalogram for guided paramedian insertion of mini-implants can prevent incisor root damage. There were no statistical differences between the distances between the implant and the roots seen in the cephalogram and all groups of CBCT images. A safety distance should be maintained to avoid a close proximity in single cases. The current investigation focuses on the anterior paramedian area of the palate. Outside that region and in complex cases with displaced teeth in the palatal area, a CBCT might be indicated.

## Data Availability

The datasets used and/or analysed during the current study are available from the corresponding author on reasonable request.

## Abbreviations

CBCT: Cone beam computed tomography
RPE: Rapid palatal expansion
TAD: Temporary anchorage device
